# Integration of bulk and single-cell transcriptomics with spatial analysis identifies candidate hub genes and inferred regulatory programs in atopic dermatitis

**DOI:** 10.3389/fimmu.2026.1863607

**Published:** 2026-09-30

**Authors:** Yuqiao Chen, Yulong Pan, Jiajie Chen, Li Lin, Shengxiu Liu

**Affiliations:** 1 Department of Dermatology, The First Affiliated Hospital of Anhui Medical University, Hefei, Anhui, China; 2 Key Laboratory of Dermatology (Anhui Medical University), Ministry of Education, Hefei, Anhui, China; 3 Inflammation and Immune-Mediated Diseases Laboratory of Anhui Province, Hefei, Anhui, China; 4 The School of Management, Soonchunhyang University, Asan-si, Chungcheongnam-do, Republic of Korea; 5 Department of Biochemistry and Molecular Biology, Metabolic Disease Research Center, School of Basic Medicine, Anhui Medical University, Hefei, Anhui, China

**Keywords:** atopic dermatitis, candidate hub genes, inferred regulatory programs, multi-omics, single-cell RNA-seq, spatial transcriptomics

## Abstract

**Background:**

Atopic dermatitis (AD) is a common chronic inflammatory disorder of the skin. Despite the availability of targeted biologics like dupilumab, heterogeneous treatment responses persist, highlighting the need for reliable biomarkers and a deeper understanding of cell-type-associated molecular programs within the tissue microenvironment.

**Methods:**

This exploratory study integrated public microarray, single-cell RNA sequencing, and spatial transcriptomic datasets. GSE130588, comprising 124 lesional AD skin samples, was used for weighted gene co-expression network analysis (WGCNA). GSE59294 comprised 40 skin biopsies from 18 patients, including 16 pre-treatment lesional, 12 post-treatment lesional, 7 pre-treatment non-lesional, and 5 post-treatment non-lesional samples, and was used for differential expression analysis. The single-cell query cohort comprised 66,846 cells from 25 samples, with downstream analyses restricted to 19 blister-derived samples. Regulon activity was computationally inferred. GSE197023 comprised 20 spatial transcriptomic samples, including 7 lesional AD, 7 non-lesional AD, and 6 healthy-control samples, and was analyzed using cell-population deconvolution. CCR7 immunoreactivity was assessed by immunohistochemistry in independent healthy-control and lesional AD skin samples (*n* = 5 per group) analyze.

**Results:**

WGCNA identified disease-severity-associated gene modules enriched in immune and cell-cycle-related processes. Nominal-*P*-value-based integration with differential expression results identified seven exploratory candidate hub genes (GZMB, CCR7, GPR183, MMP12, IL7R, RGS1, and KLHDC7B) associated with immune- cell abundance patterns. FDR-adjusted sensitivity analysis supported MMP12 and RGS1 in the lesional-versus-non-lesional comparison, whereas none of the seven genes reached nominal or FDR-adjusted significance in the paired treatment analysis. Single-cell reference mapping showed cell-type-associated expression of GZMB in cytotoxic T-cell populations and *CCR7* and *MMP12* in dendritic-cell subsets. Computational regulon analysis identified candidate cell-type-associated regulatory signals and differences in inferred regulon activity across treatment-state groups. Spatial deconvolution indicated predominantly dermal distributions of inferred immune-cell populations. Immunohistochemical assessment showed higher relative CCR7 immunoreactivity in lesional AD skin than in healthy-control skin.

**Conclusion:**

These cross-modal analyses linked candidate molecular signals todendritic-cell- and T- cell-associated patterns in AD. These findings are exploratory and hypothesis-generating and do not establish validated biomarkers, causal regulatory mechanisms, or predictors of treatment response. Independent cohort replication and functional and cell-type-specific validation are required.

## 1 Introduction

Atopic dermatitis (AD) is a frequently occurring chronic inflammatory skin condition that affects approximately 15–20% of the pediatric population and 1–3% of the adult population pediatric worldwide (1). The disease is characterized by intense pruritus, eczematous lesions, and a chronic relapsing course that significantly impairs patient quality of life (2). AD places a considerable financial burden, with annual healthcare expenditures and productivity-related losses amounting to billions of dollars in the United States alone (3).

The development of AD is driven by complex interactions among genetic susceptibility, impairment of the epidermal barrier, and dysregulated immune responses (4). Mutations in the filaggrin (FLG) gene are the predominant genetic determinant of AD susceptibility and are associated with barrier impairment and elevated transepidermal water loss (5). The immune component is dominated by type 2 inflammation, with T helper 2 (Th2) cells producing interleukin-4 (IL-4), IL-13, and IL-31, which contribute to disease pathology (6). However, recent studies have revealed additional involvement of Th17, Th22, and Th1 pathways, particularly in chronic and severe disease (7).

The advent of biologics targeting specific inflammatory pathways has revolutionized AD treatment (8). By targeting the IL-4 receptor α subunit, dupilumab, a monoclonal antibody, suppresses both IL-4- and IL-13-dependent signaling and has demonstrated significant therapeutic benefit in individuals with moderate-to-severe AD (9). However, not all patients respond adequately, and biomarkers associated with treatment response is urgently needed (10). Understanding the molecular heterogeneity of AD at the single-cell level and within the tissue microenvironment is essential for developing precision medicine approaches.

Recent developments in single-cell RNA sequencing (scRNA-seq) and spatial transcriptomics have offered new opportunities to characterize cellular diversity and tissue organization at high resolution (11). scRNA-seq studies have demonstrated heterogeneous immune and stromal cell subsets in AD skin, including distinct T cell subsets, dendritic cell populations, and inflammatory fibroblasts (12). Spatial transcriptomics adds another dimension by preserving spatial information, allowing investigation of the spatial co-occurrence of cell populations and potential microenvironmental interactions (13).

This study integrates bulk transcriptomics for identifying disease-associated gene modules, single-cell transcriptomics for characterizing cellular heterogeneity and computationally inferred candidate regulatory programs, and spatial transcriptomics to map cell-population distributions. Gene modules associated with clinical phenotypes were identified using weighted gene co-expression network analysis (WGCNA). Analysis of differential gene expression was then conducted to evaluate differences between samples derived from lesional and uninvolved skin as well as between samples collected before and after treatment. In addition, SCENIC was used to computationally infer candidate regulons and transcription factor–target relationships, while RCTD was applied to infer the spatial distribution of cell populations. Our integrative analysis identified candidate molecular signals and descriptive differences in computationally inferred regulon activity across treatment-state groups, providing an exploratory multi-omics framework for further investigation of the molecular features of AD.

## 2 Materials and methods

### 2.1 Data sources and acquisition

Publicly available transcriptomic datasets were obtained from the Gene Expression Omnibus (GEO) database (https://www.ncbi.nlm.nih.gov/geo/) (14). The GSE130588 microarray dataset, which includes 124 lesional skin samples from patients with AD, was used for weighted gene co-expression network analysis. Dataset GSE59294 was used for differential expression analysis of lesional versus non-lesional AD skin and treatment-state samples. The lesion-status comparison included 16 pretreatment lesional and 7 pretreatment non-lesional samples. The paired treatment sensitivity analysis included nine patients with matched pre- and post-dupilumab lesional skin samples; their sample accessions and treatment information are provided in **Supplementary Table S5**. Single-cell RNA sequencing data were obtained from GSE153760 and GSE158432, comprising 25 samples: 9 untreated AD samples, 5 samples collected after 16 weeks of dupilumab treatment, 4 samples collected after 1 year of dupilumab treatment, and 7 healthy- control samples (15, 16). Available sample-level demographic and clinical metadata for the single-cell RNA-seq query cohort are provided in **Supplementary Table S3**. Spatial transcriptomics data were obtained from the GEO dataset GSE197023, which included 20 skin samples: 7 lesional and 7 non-lesional skin samples from patients with AD, and 6 skin samples from healthy controls (13). A summary of the public transcriptomic datasets, sample composition, data modalities, and analytical purposes is provided in **Supplementary Table S1**.

### 2.2 Microarray data processing and WGCNA

Normalization of the raw expression data was performed using the Robust Multi-array Average (RMA) method (17). Weighted gene co-expression network analysis was carried out in R using the WGCNA package (version 1.72-1) (18). According to the scale-free topology criterion, the soft-thresholding power was selected as β = 5. Gene modules were identified using hierarchical clustering followed by dynamic tree cutting. For each module, the module eigengene was defined as the first principal component of the expression profile of its member genes. Associations between module eigengenes and disease-related clinical features, including the Eczema Area and Severity Index (EASI) and SCORing Atopic Dermatitis (SCORAD), were then assessed. Finally, genes within modules significantly associated with clinical traits were subjected to Gene Ontology (GO) enrichment analysis using the clusterProfiler package (version 4.0) (19).

### 2.3 Analysis of differential gene expression

Differential expression of genes was analyzed with the limma R package (version 3.50.0) (20). Comparisons included lesional versus non-lesional (LS vs NL) skin and pre- versus post- treatment (Pre vs Post). Genes with an absolute log2 fold change greater than 1 and a nominal p value below 0.05 were defined as differentially expressed. These exploratory thresholds were used to prioritize genes showing relatively large expression differences for downstream integrative analysis. To assess the robustness of the initial findings, Benjamini–Hochberg-adjusted *P* values were additionally examined across all genes tested in the LS-versus-NL comparison. For the treatment-associated sensitivity analysis, nine patients with matched lesional skin samples collected before and after dupilumab treatment were analyzed using two-sided paired *t* tests, followed by Benjamini–Hochberg adjustment across all 21,344 tested genes. Seven genes identified through the original nominal-*P*-value-based intersection of the two-differential expression comparisons and the WGCNA hub genes were retained as exploratory candidate hub genes for sensitivity assessment and downstream cross-modal characterization. Complete statistical results for these seven genes, including nominal and Benjamini–Hochberg-adjusted *P* values for both comparisons, are provided in **Supplementary Table S4**.

### 2.4 Single-cell RNA-seq data processing

Data derived from single-cell transcriptomic profiling were analyzed with Seurat (version 4.3.0) (21). Quality- control filtering retained cells with 200–6000 detected genesand a mitochondrial gene percentage below 20%. These criteria were applied to exclude low-complexity or potentially damaged cells, as well as cells with unusually high feature counts that might represent multiplets. Normalization was carried out with SCTransform. Variable features were selected and subsequently subjected to dimensionality reduction using PCA followed by UMAP. Cell type annotation was performed using Symphony mapping to the DISCO skin reference atlas (22). To reduce heterogeneity associated with different sampling methods, downstream cell-type analyses were restricted to blister-derived samples. Only subclusters containing more than 200 cells were retained to reduce instability associated with very small cell populations.

### 2.5 SCENIC analysis

Candidate transcriptional regulatory networks were computationally inferred using pySCENIC (version 0.12.1) (23). Meta-cells were generated to decrease computational complexity and enhance the signal-to-noise ratio. The analytical workflow comprised three steps: (1) construction of gene regulatory networks with GRNBoost2, (2) identification of regulons with cisTarget based on motif databases, and (3) evaluation of regulon activity with AUCell. Regulon specificity scores (RSSs) were calculated for each cell type using Jensen-Shannon divergence. Comparisons of inferred regulon activity among untreated, 16-week, and 1-year treatment-state groups were performed as exploratory descriptive analyses. Because samples collected at different time points could originate from the same patients, these comparisons were not interpreted as independent patient-level treatment effects, and cells or meta-cells were not considered independent clinical replicates. All regulons, transcription factor–target relationships, and activity patterns identified by SCENIC were treated as computational predictions rather than experimentally validated regulatory mechanisms.

### 2.6 Spatial transcriptomics analysis

Spatial transcriptomics data were processed using Seurat (21). Spatial spots with more than 300 detected features and more than 500 total counts were retained. These filtering criteria were applied to exclude low-information spots with insufficient transcriptomic complexity for downstream clustering and cell-type deconvolution. Data were normalized using SCTransform and clustered using the Louvain algorithm. Cell-type deconvolution was carried out with RCTD (Robust Cell Type Decomposition) and the single-cell reference dataset (24) to infer spatial cell-population distributions in AD and healthy-control skin tissue. RCTD estimates the relative contributions of reference cell populations to individual spatial spots; therefore, the resulting maps were interpreted as inferred cell-population distributions rather than direct evidence of cellular co-localization, interaction, migration, or communication. The same preprocessing and deconvolution workflow was applied to all spatial samples. Representative sections displayed in the main figure were selected after quality control based on adequate tissue coverage, sufficient retained spots, and interpretable deconvolution maps; their selection was solely for visualization, and no statistical inference was based on the representative sections alone.

### 2.7 Analysis of immune cell infiltration

Relative abundance of infiltrating immune and stromal cell subsets was estimated using the MCP-counter (Microenvironment Cell Populations counter) algorithm (25). This method provides quantitative estimates for eight immune cell populations, including T cells, CD8+ T cells, cytotoxic lymphocytes, NK cells, B-lineage cells, monocytes/macrophages, myeloid dendritic cells, and neutrophils, as well as two stromal components: endothelial cells and fibroblasts. Associations between candidate hub- gene expression and the estimated abundance of immune- cell populations were evaluated using Spearman correlation analysis.

### 2.8 Immunohistochemical assessment

To assess the immunoreactivity of a selected candidate hub gene, immunohistochemistry (IHC) was performed on independent lesional skin samples from patients with AD and healthy-control skin samples (n = 5 biological samples per group). These tissue samples were independent of the public GEO datasets used for the transcriptomic analyses. Tissue sections were stained with an antibody against CCR7 (catalog no. R380950). The antibody dilution was not available in the archived experimental records and therefore could not be reported. The staining signals were assessed independently by two pathologists who were blinded to the clinical data. Quantitative data were available as relative CCR7 immunoreactivity values for five healthy-control and five lesional AD skin samples. The values were analyzed as provided in the available experimental records and did not represent an H-score or a count of positive cells per mm². Cell-type-specific co-localization staining was not performed; therefore, the immunohistochemical assessment could not determine the cellular source of CCR7 immunoreactivity.

### 2.9 Statistical analysis

Statistical analyses were conducted primarily in R (version 4.2.2). The paired treatment sensitivity analysis was performed in Python 3.12.14 using SciPy 1.17.0, with Benjamini–Hochberg adjustment applied across all tested genes. Continuous variables were summarized as mean ± standard deviation (SD). For two-group comparisons, either the independent-samples t-test or the nonparametric Mann-Whitney U test was applied, depending on the distribution of the data. For the CCR7 immunohistochemical assessment, relative immunoreactivity values were compared between the healthy-control and lesional AD groups using the exact two-sided Mann–Whitney U test. Associations among variables were examined using Spearman correlation analysis. Unless otherwise specified, a two-sided nominal *P* value below 0.05 was used as the exploratory significance threshold. Benjamini–Hochberg-adjusted *P* values were additionally calculated for the differential expression sensitivity analyses described in Section 2.3.

### 2.10 Computational reproducibility

Available analysis scripts, including the code used for the paired treatment sensitivity analysis, are provided as **Supplementary Code S1**. The accompanying README describes the purpose and execution order of the included scripts. However, because some original environment configurations, reference resources, and intermediate objects were unavailable, the deposited materials do not constitute a fully executable end-to-end workflow.

## 3 Results

**Figure 1** presents the technical roadmap and overall workflow of the study.

**Figure 1:**
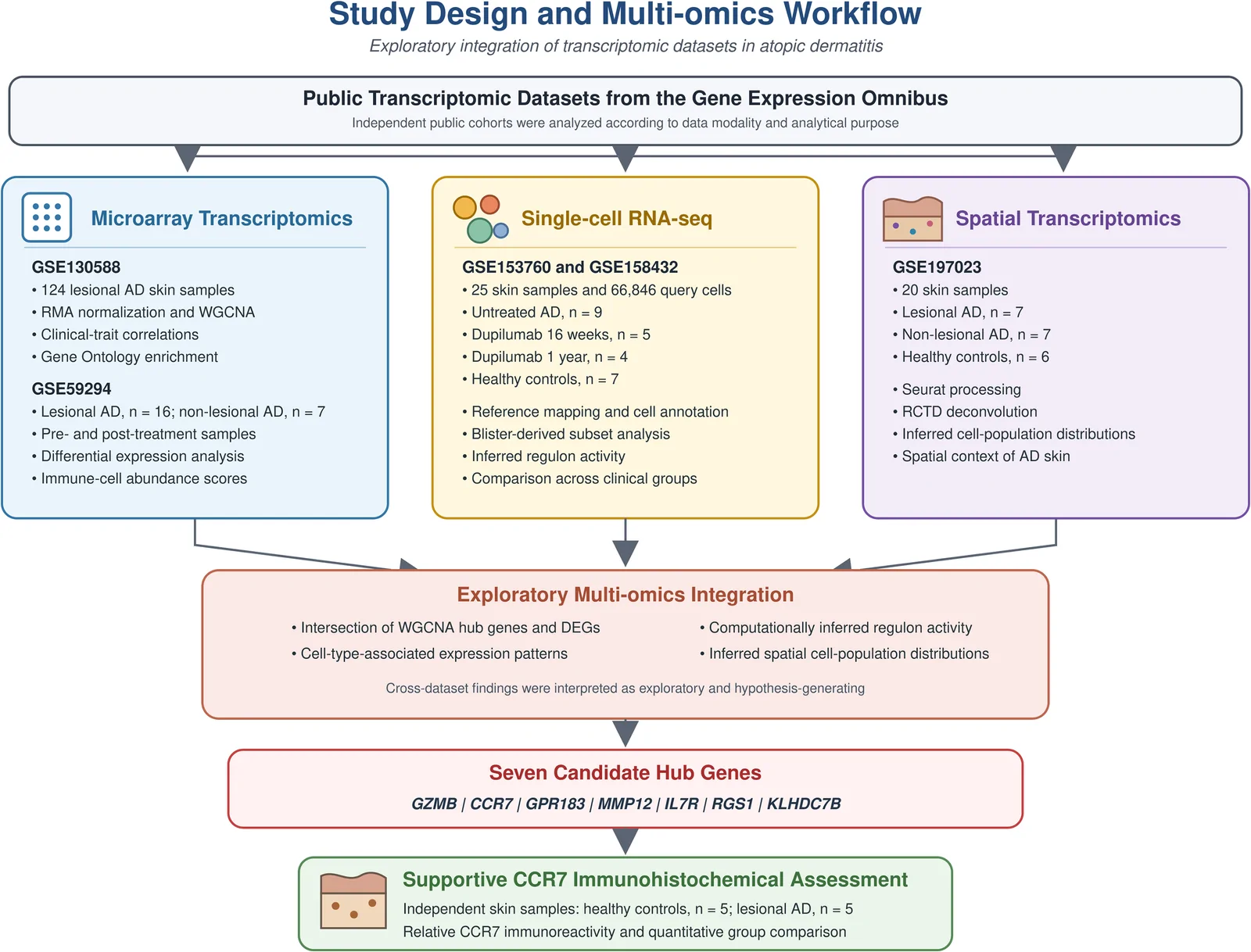
Study design and multi-omics workflow. Overview of the exploratory integration of public microarray, single-cell RNA-seq, and spatial transcriptomic datasets in AD. GSE130588 and GSE59294 were used for WGCNA, differential expression analysis, and immune-cell abundance estimation; GSE153760 and GSE158432 were used for reference mapping, cell-type annotation, and computational inference of regulon activity; and GSE197023 was used for RCTD-based inference of spatial cell-population distributions. The integrated analyses identified seven candidate hub genes, followed by supportive immunohistochemical assessment of CCR7 in independent skin samples.

### 3.1 WGCNA identifies disease-associated gene modules

Co-expression network analysis of 124 lesional skin samples derived from individuals with AD revealed multiple gene modules that were significantly associated with clinical parameters. A soft-thresholding power of β = 5 was selected based on the scale-free topology criterion (**Figure 2A**). Hierarchical clustering delineated distinct gene modules, with the green and brown modules showing significant positive correlations with disease severity scores (EASI: r = 0.49, p < 0.001and r = 0.55, p < 0.001; SCORAD: r = 0.52, p < 0.001 and r = 0.57, p < 0.001). In contrast, the greenyellow and grey60 modules showed negative correlations with disease severity (**Figures 2B, C**). Functional enrichment analysis based on Gene Ontology revealed distinct biological features of the two modules: the green module was linked to immune-related processes such as leukocyte adhesion and T-cell activation, while the brown module was mainly associated with cell-cycle pathways (**Figure 2D**). Together, these results identified disease-severity-associated gene modules with distinct immune- and cell-cycle-related molecular patterns in AD lesions.

**Figure 2:**
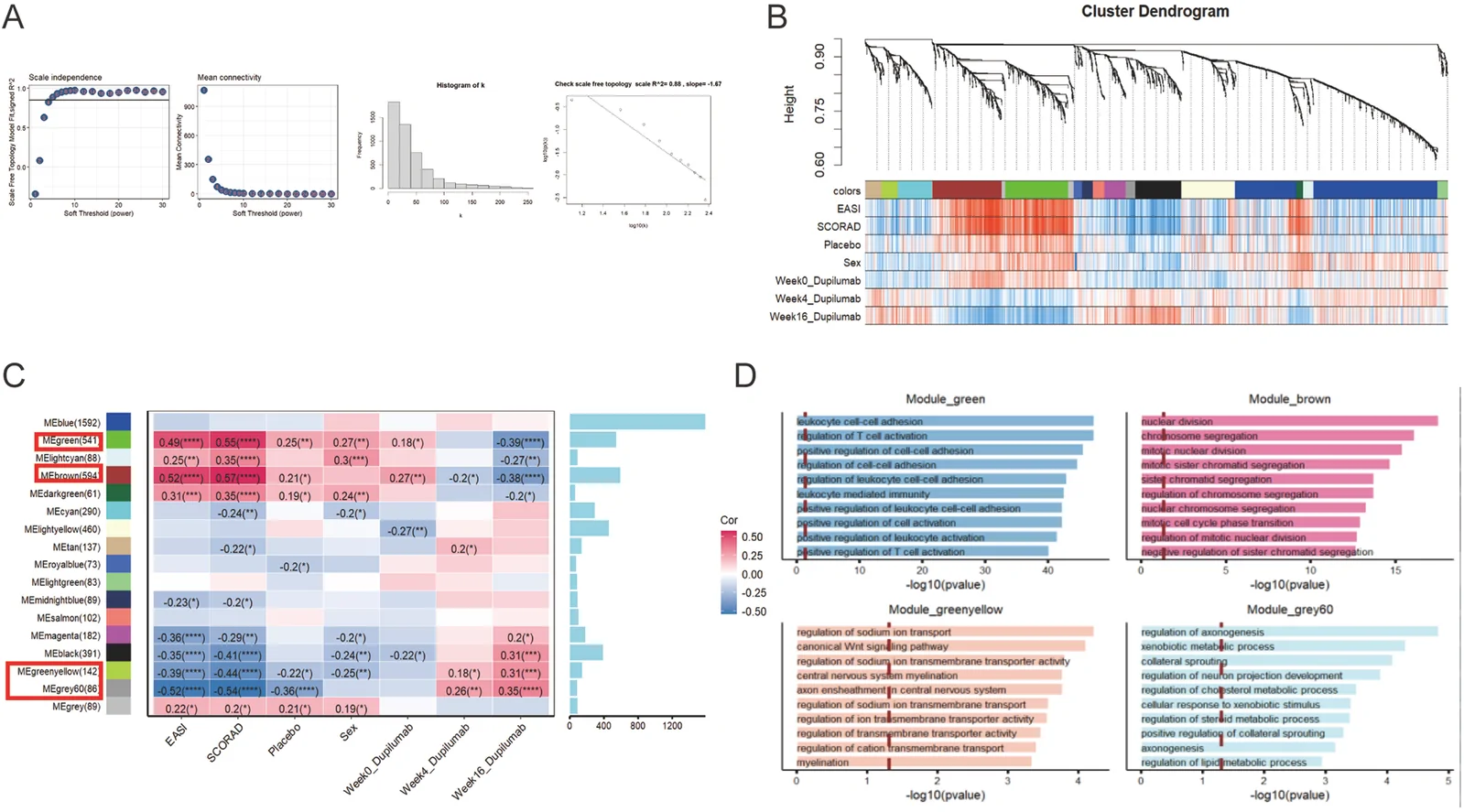
WGCNA of lesional AD skin transcriptomic data. **(A)** Selection of the soft-thresholding power based on scale-free topology and mean connectivity; β = 5 was selected for network construction. **(B)** Hierarchical clustering dendrogram of the samples with the corresponding clinical-trait heatmap. **(C)** Heatmap showing correlations between module eigengenes and clinical traits. Correlation coefficients and nominal P values are displayed within the cells. **(D)** Gene Ontology enrichment results for the green, brown, greenyellow, and grey60 modules. The analysis included 124 lesional AD skin samples; *n* denotes biological skin samples rather than technical replicatesmeter grey. *P < 0.05, **P < 0.01, ***P < 0.001, and ****P < 0.0001.

### 3.2 Differential expression and sensitivity analyses of lesional and treatment-state samples

Exploratory differential expression analysis comparing lesional (LS) and non-lesional (NL) skin identified 246 genes with increased expression and 92 genes with reduced expression using an absolute log2FC greater than 1 and a nominal *P* value below 0.05 (**Figures 3A, B**). Pathway enrichment analysis indicated that genes with elevated expression were mainly associated with inflammatory signaling processes, particularly cytokine–cytokine receptor interaction and the JAK–STAT signaling pathway. In the paired sensitivity analysis of nine patients with matched lesional skin samples collected before and after dupilumab treatment (**Supplementary Table S5**), 49 genes met the original exploratory thresholds of a nominal P value below 0.05 and an absolute mean paired expression difference greater than 1, including 35 genes with higher expression before treatment and 14 genes with higher expression after treatment (**Figure 3B**). However, no gene met the combined criterion of an FDR below 0.05 and an absolute mean paired difference greater than 1. These findings characterized lesion-associated expression differences but indicated that the treatment-state-associated findings did not remain robust after paired-sample analysis and multiple-testing correction.

**Figure 3:**
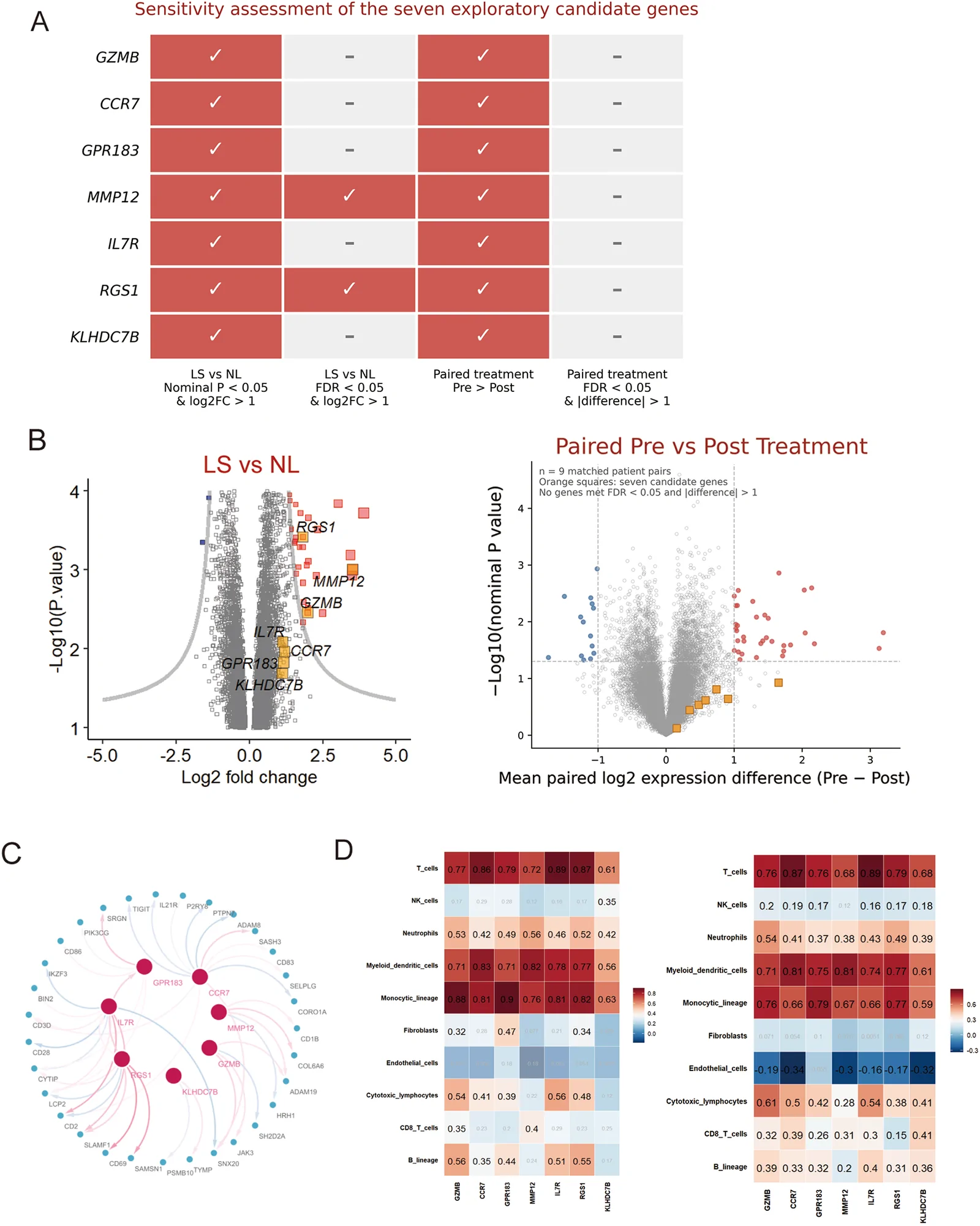
Differential Expression, Candidate- Gene Sensitivity Analysis, and Immune-Cell Abundance Analysis. **(A)** Sensitivity matrix summarizing the statistical support for the seven exploratory candidate hub genes in the lesional versus non-lesional comparison and the paired pre- versus post-treatment analysis. Check marks indicate that the corresponding criterion was met, whereas dashes indicate that it was not met. Complete nominal and FDR-adjusted statistical results are provided in **Supplementary Table S4**. **(B)** Volcano plots for the lesional versus non-lesional comparison (16 lesional and 7 non-lesional AD skin samples) and the paired pre- versus post-treatment analysis (9 matched patient pairs; **Supplementary Table S5**). In the paired treatment volcano plot, the horizontal dashed line indicates a nominal P value of 0.05, and the vertical dashed lines indicate mean paired log2 expression differences of −1 and 1.Red and blue points indicate genes meeting these exploratory thresholds with higher expression in lesional or pretreatment samples and non-lesional or post-treatment samples, respectively; orange squares indicate the seven exploratory candidate hub genes. **(C)** Network showing the seven exploratory candidate hub genes and their associated genes in the green WGCNA module. **(D)** Spearman correlation heatmaps showing associations between exploratory candidate hub-gene expression and MCP-counter-derived immune-cell abundance scores in GSE130588 and GSE59294. Values within the heatmaps represent Spearman correlation coefficients; red and blue indicate positive and negative correlations, respectively, with color intensity indicating the magnitude of the correlation. Each correlation analysis was performed at the biological-sample level; no technical replicates were treated as independent observations.

### 3.3 Exploratory candidate hub genes identified through integrative analysis

The original nominal-*P*-value-based integration of WGCNA hub genes and differential expression results identified seven exploratory candidate hub genes: GZMB, CCR7, GPR183, MMP12, IL7R, RGS1, and KLHDC7B. Complete nominal and FDR-adjusted statistical results for these genes are provided in **Supplementary Table S4**. In the FDR-adjusted sensitivity analysis, MMP12 and RGS1 met the combined criterion at FDR < 0.05 with log2FC > 1 in the LS-versus-NL comparison, whereas the other five genes did not meet the FDR-adjusted criterion. In the paired treatment analysis, all seven genes showed higher mean expression before treatment than after treatment, but none reached nominal P < 0.05 or FDR < 0.05 (**Figure 3A**; **Supplementary Figure S1**; **Supplementary Table S4**). GZMB (granzyme B) is a serine protease predominantly produced by cytotoxic T lymphocytes and NK cells and plays an important role in inducing apoptosis of target cells (26). CCR7 (C-C chemokine receptor 7) is involved in directing the trafficking of T cells and dendritic cells toward lymphoid tissues and serves a key role in immune-cell trafficking during inflammatory responses (27). GPR183 (G protein-coupled receptor 183, also referred to as EBI2) guides immune- cell positioning through oxysterol sensing (28). MMP12 (matrix metalloproteinase-12) is an elastase produced by macrophages involved in tissue remodeling (29). IL7R (interleukin-7 receptor) is essential for T- cell development and homeostasis (30). RGS1 (regulator of G protein signaling 1) modulates chemokine receptor signaling and may influence T- cell migration (31). KLHDC7B (kelch domain containing 7B) is a less characterized gene potentially involved in protein ubiquitination. Given the limited FDR-adjusted support for the complete seven-gene set, these genes were retained only as exploratory candidate hub genes for cross-modal characterization; their gene-association network is shown in **Figure 3C**.

### 3.4 Immune cell abundance scores correlate with candidate hub gene expression

MCP-counter analysis revealed that the expression levels of all seven candidate hub genes were markedly correlated with specific immune- cell abundance scores in two bulk transcriptomic datasets (**Figure 3D**). GZMB expression was correlated with T-cell abundance scores (r = 0.77, P < 0.001; r = 0.76, P < 0.001) and cytotoxic-lymphocyte abundance scores (r = 0.54, P < 0.001; r = 0.61, P < 0.001).CCR7 expression was correlated with monocytic- lineage cell abundance scores (r = 0.81, p < 0.001; r = 0.66, p < 0.001) and myeloid dendritic- cell abundance scores (r = 0.83, p < 0.001; r = 0.81, p < 0.001). MMP12 expression showed strong correlations with monocytic-lineage cell abundance scores (r = 0.76, P < 0.001; r = 0.67, P < 0.001), consistent with its known expression in macrophage-lineage cells (**Figure 3D**).These correlations indicate that the candidate hub genes are associated with distinct immune-cell abundance patterns in AD.

### 3.5 Single-cell atlas reveals cellular heterogeneity in AD skin

To obtain a consistent cell-type annotation framework across multi-cohort skin scRNA-seq data, we first constructed a skin single-cell reference atlas. Reference data were collected from DISCO and comprised 394,594 cells from 20 publicly available datasets (e.g., GSE163973, GSE132802, GSE151177) (**Figures 4A–C**). After standard quality control, normalization, and PCA, we performed cross-dataset integration in PCA space using Harmony to mitigate batch effects, generating a unified reference model and its low-dimensional embedding (refUMAP) for downstream query projection and label transfer. We then performed query-to-reference mapping for the study data. The query comprised 66,846 cells from four clinical/experimental groups: Untreated AD (n = 9), Dupilumab 16w (n = 5), Dupilumab 1 year (n = 4), and HC (n = 7). Using the Symphony reference mapping workflow, query cells were projected onto the reference latent space and annotated via label transfer under default prediction/confidence settings. The mapped refUMAP showed that query cells aligned with the major lineage architecture of the skin reference atlas, forming compartments corresponding to Keratinocytes, Fibroblasts, Endothelial cells (EC), Smooth muscle cells (SMC), and immune populations including Myeloid, B & Plasma, and NK & T cells. In addition to these major classes, multiple finer subpopulations defined in the reference were identified in the query data, as indicated by the legend. To assess comparability across conditions within a shared coordinate system, we visualized each group on the same refUMAP. All groups populated the major reference-defined compartments, indicating that the mapping captured the major cell-lineage compartments across disease and treatment states. Notably, local density distributions differed across groups in immune-associated regions, such as the Myeloid and NK/T compartments, motivating subsequent quantitative comparisons of cell-type composition and cell-type–resolved transcriptional changes using the unified reference-based annotations.

**Figure 4:**
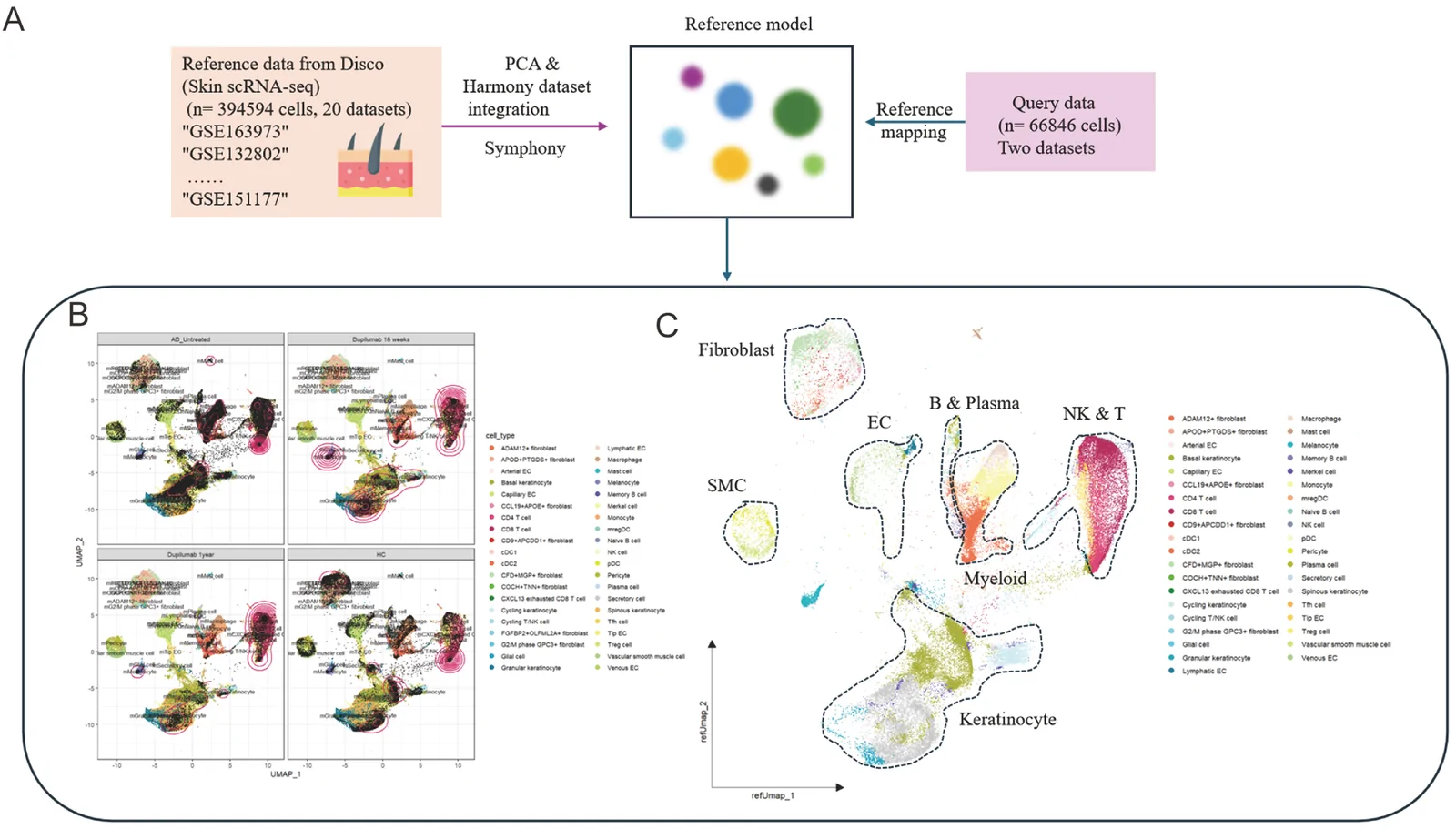
Construction of the skin single-cell reference atlas and mapping of the query scRNA-seq data. **(A)** Schematic of the reference-mapping workflow. The DISCO skin reference atlas comprised 394,594 cells from 20 publicly available datasets and was integrated using Harmony to construct the reference model. The query dataset comprised 66,846 cells from 25 biological samples obtained from GSE153760 and GSE158432. **(B)** Reference-mapped UMAPs of the query cells stratified by clinical group: untreated AD (*n* = 9), dupilumab treatment for 16 weeks (*n* = 5), dupilumab treatment for 1 year (*n* = 4), and healthy controls (*n* = 7). **(C)** Combined reference-mapped UMAP showing the major skin cell lineages and transferred cell-type annotations. Each point represents one cell. Sample numbers refer to biological samples; no technical replicates were treated as independent samples color.

Comparison among individuals with untreated AD, treated AD, and healthy controls characterized the cellular landscape of AD skin, although no significant differences in cell-type proportions were detected across the groups. Additionally, to minimize potential bias in cell-type composition introduced by the sampling method (blister-derived cells vs biopsy), we restricted downstream analyses to blister-derived samples and further retained only subclusters containing more than 200 cells analyze. After this filtering, single-cell RNA-seq analysis identified 14 distinct cell types in AD skin, including T cell subsets (CD4+, CD8+, Tfh, Treg, cycling T/NK), myeloid cells (cDC2, mregDC, monocytes), epidermal cells (basal, spinous, granular, cycling keratinocytes), and other cell types (melanocytes, Merkel cells) (**Figure 5A**). Hub gene expression analysis at single-cell resolution revealed cell type-specific patterns: GZMB was primarily expressed in CD8+ T cells and cycling T/NK cells, CCR7 in mreg DCs, and MMP12 in mregDCs and cDC2 cells (**Figures 5B–D**). These findings provided cell-type context for the candidate hub genes without establishing their cell-specific functions.

**Figure 5:**
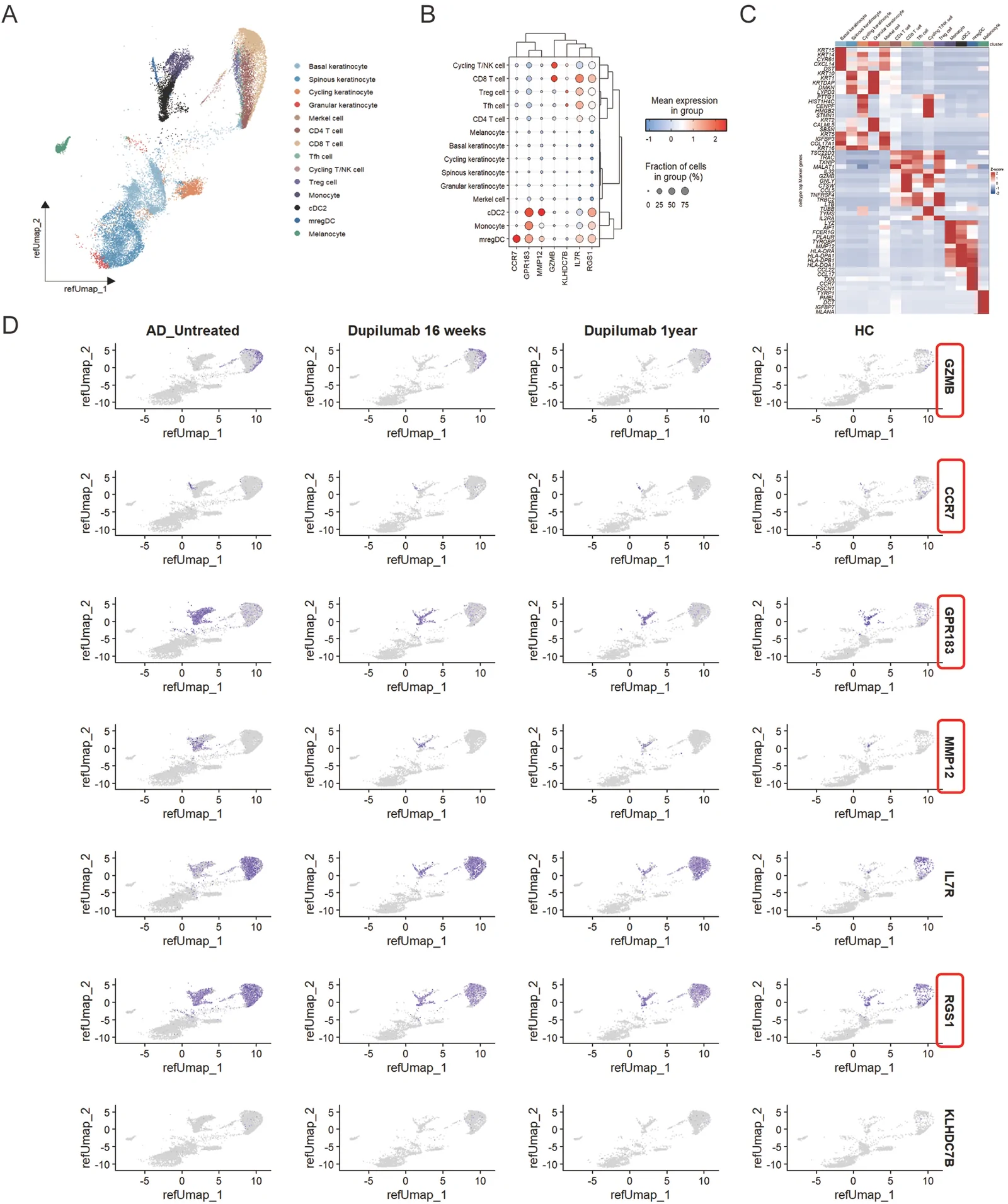
Cell-type annotation and hub gene expression patterns inanalyze blister-derived single-cell samples. Analyses were restricted to blister-derived samples from untreated AD (*n* = 5), dupilumab treatment for 16 weeks (*n* = 5), dupilumab treatment for 1 year (*n* = 4), and healthy controls (*n* = 5), comprising 19 biological samples in total. **(A)** Reference-mapped UMAP showing the annotated cell populations. Each point represents one cell. **(B)** Dot plot showing candidate hub-gene expression across cell populations. Dot size represents the proportion of cells expressing the gene, and color represents the scaled mean expression level. **(C)** Heatmap showing representative marker-gene expression across the annotated cell populations. **(D)** Feature plots showing the expression patterns of the seven candidate hub genes across the four clinical groups. Sample numbers refer to biological samples; no technical replicates were treated as independent samples.

### 3.6 SCENIC inference of cell- type-associated transcriptional programs

SCENIC was used to computationally infer patterns of regulon activity across the annotated cell populations (**Figure 6**). Regulon specificity score (RSS) analysis revealed cell type-specific patterns of inferred regulon activity. In mature regulatory dendritic cells (mregDCs), the computationally inferred CDX2 and KLF7 regulons showed relatively high specificity, suggesting candidate cell-type-associated regulatory signals. Given the limited prior evidence supporting a regulatory role for CDX2 in mregDCs, the CDX2 signal should be regarded as a hypothesis-generating computational prediction requiring independent validation. In conventional type 2 dendritic cells (cDC2), ZNF366 and SPIB regulons showed relatively specific activity, in line with the reported function of SPIB in dendritic cell development (32). CD8+ T cells showed relatively specific activity of GFI1B and EOMES regulons, which have been implicated in cytotoxic T cell differentiation (33). Cycling T/NK cells exhibited relatively specific activity of ZNF410 and BRCA1 regulons, consistent with their association with cell- proliferation programs. Selected regulons, including CDX2, IRF8, and KLF7, showed descriptive differences in computationally inferred activity across untreated AD, 16-week dupilumab, 1-year dupilumab, and healthy-control groups. Because patient-level clinical response outcomes were incomplete and samples obtained at different time points could be dependent, these patterns were not interpreted as patient-level treatment responses or independent treatment effects. Overall, the SCENIC analysis identified candidate cell-type-associated regulon signals that require independent molecular and functional validation.

**Figure 6:**
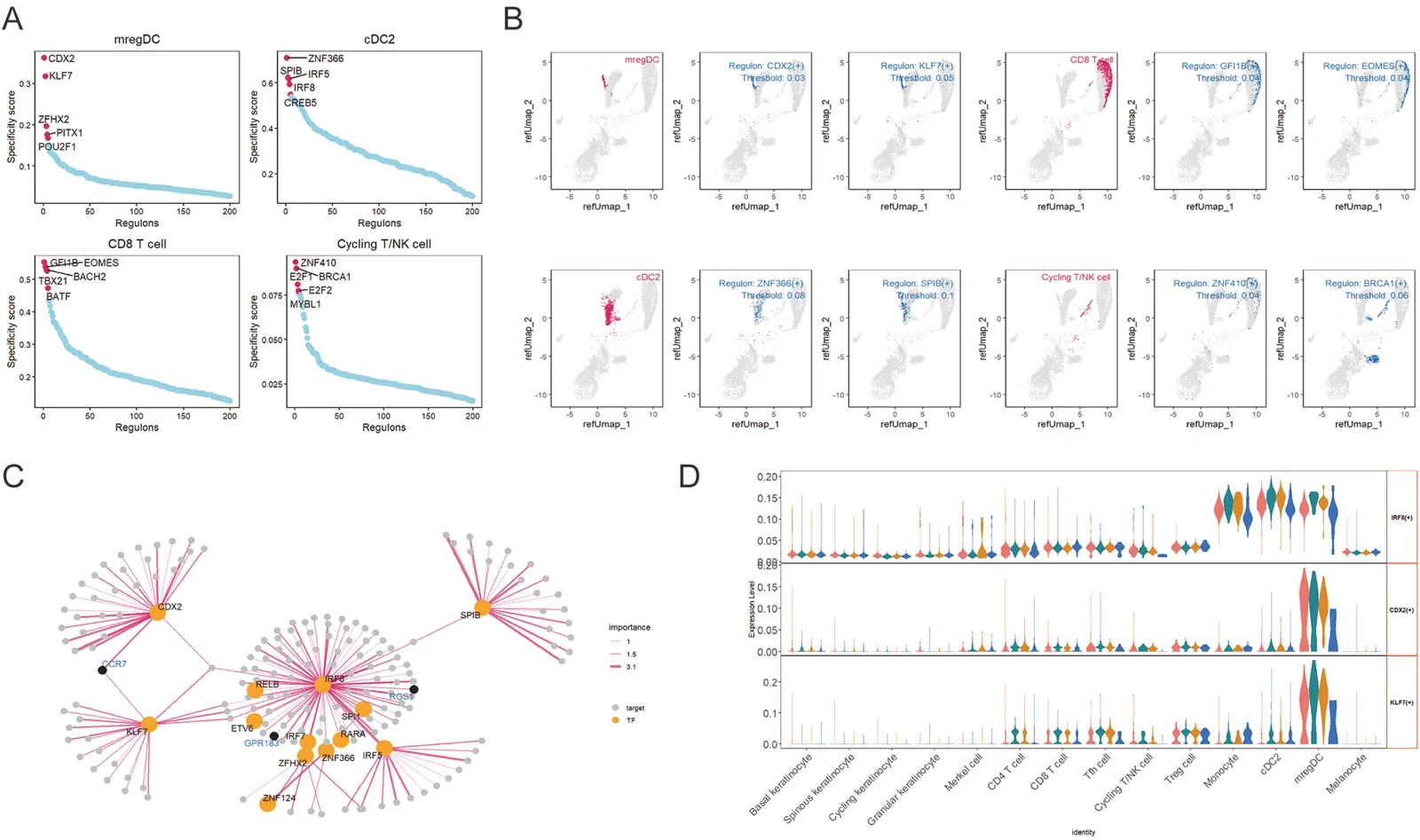
Computational inference of cell-type-associated regulon activity using SCENIC. The analysis was performed using cells from 19 blister-derived biological samples: untreated AD (*n* = 5), dupilumab treatment for 16 weeks (*n* = 5), dupilumab treatment for 1 year (*n* = 4), and healthy controls (*n* = 5). **(A)** Regulon specificity score ranking plots for mregDCs, cDC2s, CD8+ T cells, and cycling T/NK cells. **(B)** Reference-mapped UMAPs showing the computationally inferred activity of selected regulons in the corresponding cell populations. Each point represents one cell. **(C)** Computationally inferred transcription factor–target networks. Orange nodes represent transcription factors, grey nodes represent predicted target genes, and edge intensity represents the relative importance assigned by the inference model. **(D)** Violin plots showing the distributions of inferred IRF8, CDX2, and KLF7 regulon activity across cell populations and clinical groups. Violin width represents cell-density distribution rather than measurement uncertainty; no error bars are displayed. Sample numbers refer to biological specimens rather than necessarily independent participants, because specimens collected at different time points could originate from the same patients. Cells and meta-cells were not treated as independent clinical replicates, and the displayed treatment-state differences were interpreted descriptively rather than as patient-level treatment responses or independent treatment effects. All regulons, transcription factor–target relationships, and activity patterns shown in this figure represent computational predictions and should not be interpreted as experimentally validated regulatory mechanisms.

### 3.7 RCTD inference of spatial cell- population distributions

RCTD deconvolution was used to infer spatial cell-population distributions across the analyzed AD and healthy-control skin sections, with representative sections shown in **Figure 7A, B**. In the displayed representative sections, T-cell- and myeloid-cell-associated signals showed higher inferred RCTD weights in dermal regions, whereas keratinocyte-associated signals were distributed predominantly in epidermal regions. RCTD weight maps also showed inferred spatial patterns of mregDC-like and cDC2-like populations associated with CCR7 and MMP12 expression, respectively. These computational results provide tissue-level context for the cell-type-associated transcriptomic findings but do not demonstrate cellular co-localization, direct cell–cell interaction, immune-cell migration, or functional communicationwhile program modeling.

**Figure 7:**
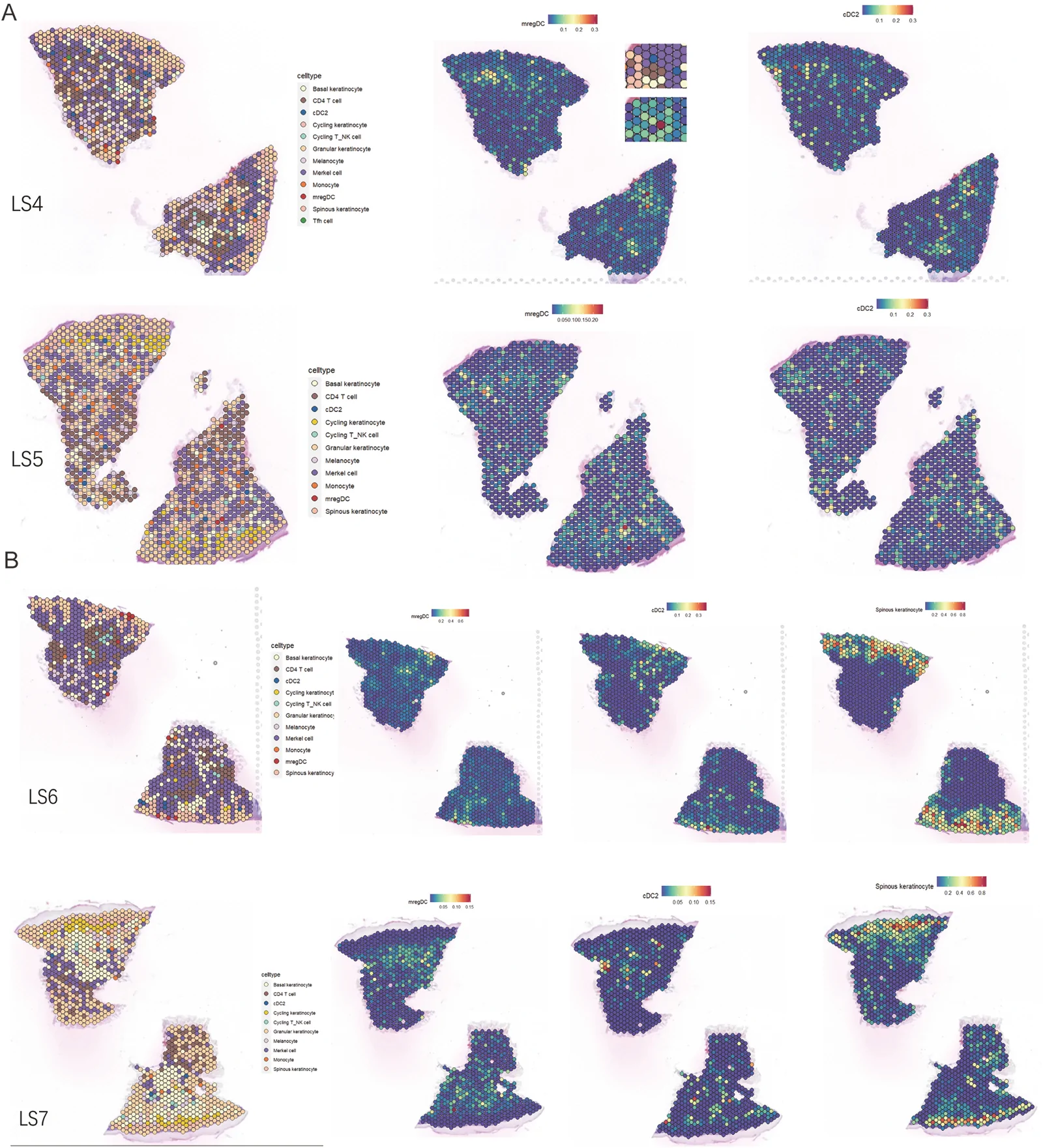
RCTD-inferred spatial distributions of cell populations in AD skin. The GSE197023 spatial transcriptomic dataset comprised 20 biological skin samples: 7 lesional AD, 7 non-lesional AD, and 6 healthy-control samples. Representative spatial maps from four lesional AD samples—LS4 (GSM5907080), LS5 (GSM5907081), LS6 (GSM5907082), and LS7 (GSM5907083)—are shown. These sections were selected after quality control based on adequate tissue coverage, sufficient retained spots, and interpretable deconvolution maps and were used for visualization only; no statistical inference was based on these sections alone. **(A)** RCTD-inferred cell-population distributions and weight maps for mregDC-like and cDC2-like populations in LS4 and LS5. **(B)** RCTD-inferred cell-population distributions and weight maps for mregDC-like, cDC2-like, and spinous-keratinocyte populations in LS6 and LS7. Each hexagonal point represents one spatial transcriptomic capture spot, and the color intensity in the weight maps represents the RCTD-inferred relative contribution of the corresponding reference cell population. These maps represent computationally inferred cell-population distributions and do not demonstrate cellular co-localization, cell migration, direct cell–cell interaction, or functional communication. Sample numbers refer to biological samples; no technical replicates were treated as independent samples.

### 3.8 Immunohistochemical assessment of CCR7

Immunohistochemical assessment provided supportive but limited evidence related to the transcriptomic findings. As shown in **Figure 8A**, CCR7 immunoreactivity was observed in both healthy-control and lesional AD skin, including diffuse staining in the epidermal and stratum corneum regions as well as staining in the dermis. Because this pattern may include nonspecific background and co-localization with immune-cell markers was not performed, the IHC findings were not used to assign CCR7 to a specific cell population or subcellular compartment. Quantitative analysis showed higher relative CCR7 immunoreactivity in lesional AD skin than in healthy-control skin (*n* = 5 independent biological samples per group; exact two-sided Mann–Whitney U test, *P* = 0.0317) (**Figure 8B**). Individual measurements are provided in **Supplementary Table S2**. These findings should be interpreted as supportive immunohistochemical evidence and require confirmation using appropriate staining controls, cell-type-specific co-localization, and larger independent cohortsmodeling.

**Figure 8:**
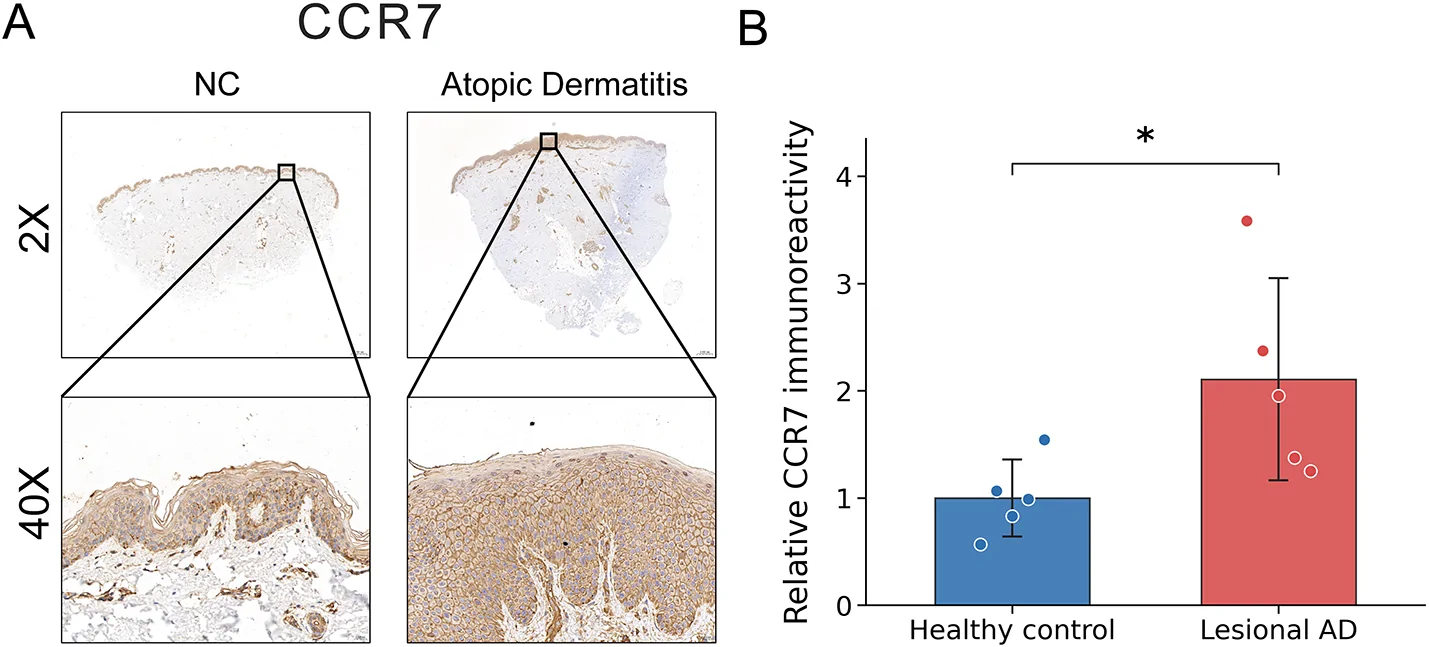
Immunohistochemical assessment and quantitative analysis of CCR7 in healthy-control and lesional AD skin. **(A)** Representative immunohistochemical images of CCR7 staining in healthy-control skin and lesional AD skin at 2× and 40× magnification. The boxed regions in the 2× images correspond to the areas shown at 40× magnification. **(B)** Relative CCR7 immunoreactivity in healthy-control and lesional AD skin samples (*n* = 5 independent biological samples per group). Each point represents one biological skin sample, and bars and error bars represent mean ± SD. Group differences were assessed using the exact two-sided Mann–Whitney U test (*P* = 0.0317). Technical replicates were not treated as independent observations. Individual quantitative measurements are provided in **Supplementary Table S2**. Diffuse epidermal and stratum corneum staining may include nonspecific background. Accordingly, the staining pattern was not used to infer the cell-type-specific or subcellular localization of CCR7. NC denotes healthy-control skin. * indicates P < 0.05.

## 4 Discussion

This exploratory multi-omics study integrated microarray, single-cell RNA-seq, and spatial transcriptomic analyses to characterize AD-associated molecular signals across gene-module, cell-type, and tissue-distribution levels. The principal contribution of this study does not lie in presenting the individual genes as entirely novel biomarkers or demonstrating their diagnostic superiority. Instead, it lies in the cross-modal characterization of seven exploratory candidate hub genes (*GZMB, CCR7, GPR183, MMP12, IL7R, RGS1*, and *KLHDC7B*) in relation to disease-severity-associated gene modules, immune-cell abundance scores, cell-type-associated expression patterns, computationally inferred regulon activity, and inferred spatial cell-population distributions. The FDR-adjusted sensitivity analysis supported MMP12 and RGS1 in the lesional-versus-non-lesional comparison but did not support the robustness of the complete seven-gene set. Moreover, none of the seven genes reached nominal or FDR-adjusted statistical significance in the paired treatment analysis. Accordingly, the seven-gene findings should be regarded as exploratory and hypothesis-generating and require confirmation in independent datasets.

The candidate hub genes identified in this investigation have either well-documented or emerging functions in cutaneous inflammatory disorders. GZMB, a product of NK cells and cytotoxic T lymphocytes, has been linked to tissue injury across a spectrum of autoimmune diseases (26). Earlier evidence suggests that lesional skin from patients with AD contains higher GZMB transcript levels in CD4+ and CD8+ T-cell populations than healthy control skin. Histamine stimulation via the H2 receptor has also been reported to increase GZMB expression in CD4+ T cells at both the transcript and protein levels (34). The observed increase in GZMB within AD lesions is consistent with the possible involvement of cytotoxic effector mechanisms in AD.

CCR7 plays an important role in directing the homing of dendritic cells and the lymphatic trafficking of T lymphocytes (27). The CCR7–CCL19/CCL21 signaling axis contributes to the migration of mature dendritic cells from peripheral tissues to draining lymphoid organs, thereby facilitating antigen presentation to naïve T cells (35). Based on its established biological functions, increased CCR7 expression in AD could hypothetically be related to dendritic- cell and T- cell trafficking in inflamed skin. However, the present RCTD analysis did not directly assess immune-cell recruitment or migration, and this possibility requires independent experimental investigation.

The oxysterol receptor GPR183 (EBI2) contributes to immune cell positioning and migration (28). GPR183 signaling is important for the proper positioning of B cells, T cells, and dendritic cells within lymphoid tissues and peripheral tissues. Altered GPR183 expression in AD may be relevant to immune- cell positioning and persistent inflammation in chronic AD lesions; however, the present analysis does not establish altered cellular localization or migration.

MMP12, a macrophage-derived elastase, is involved in extracellular matrix remodeling and tissue damage (29). Recent evidence has shown that MMP12 activity is elevated in AD lesions and is primarily attributed to infiltrating M2 macrophages in lesional skin (36). Histamine and Th2 cytokines enhance MMP12 expression during monocyte differentiation toward an M2 macrophage phenotype, whereas this induction may be suppressed by dupilumab treatment (36). Our findings are consistent with a potential role for MMP12 in tissue remodeling modeling in chronic AD lesions.

The identification of CDX2 and KLF7 regulons in mregDCs provides exploratory evidence of candidate cell-type-associated regulatory signals in AD. Although CDX2 has been linked to intestinal immune regulation and barrier maintenance (37), evidence supporting a functional role for CDX2 in cutaneous mregDCs is currently lacking. Given the co-expression- and motif-based nature of SCENIC, the CDX2 signal may represent a false-positive inference or computational artifact. It should therefore not be interpreted as evidence that CDX2 directly regulates dendritic-cell identity or function without independent experimental validation while program.

The identification of ZNF410 and BRCA1 regulons in cycling T/NK cells is consistent with the activation of cell-cycle-related transcriptional programs in this populationin AD. BRCA1 is widely recognized for its role in DNA repair and cell-cycle checkpoint control (38), and its inferred activity in cycling T/NK cells is consistent with a proliferative state in this population in AD skin.

Descriptive differences in inferred regulon activity across treatment-state groups, particularly those involving IRF8, CDX2, and KLF7, suggest variation in computationally inferred transcriptional programs among the analyzed groups. Given the incomplete patient-level clinical response outcomes and potential within-patient dependence across sampling time points, these findings should be interpreted as exploratory computational inferences rather than evidence of direct regulatory mechanisms, independent treatment effects, or patient-level treatment responses. IRF8 is involved in the development and functional specification of dendritic cells signaling (39). In our SCENIC analysis, the CDX2 and KLF7 regulons showed relatively specific computationally inferred activity in mregDCs; however, particularly for CDX2, these signals represent computational predictions and do not establish a regulatory role in mregDCs. These findings generate hypotheses regarding treatment-state-associated transcriptional programs in AD, which require independent molecular and functional validation.

Spatial transcriptomics provided tissue-level context for the single-cell findings. In the representative lesional AD sections, RCTD deconvolution indicated distinct spatial distribution patterns of mregDC-like and cDC2-like populations associated with CCR7 and MMP12 expression, respectively. However, these computationally inferred distributions do not establish cellular co-localization, direct cell–cell interactions, immune-cell migration, or functional communication. Future studies should combine spatial co-localization analysis with ligand–receptor communication frameworks, such as CellChat or NicheNet, and independent experimental validation to investigate these potential relationshipsmodeling.

Integrating bulk, single-cell, and spatial transcriptomic approaches enabled a multilayered characterization of the molecular features of AD. WGCNA of bulk transcriptomics data identified gene modules associated with disease severity, while differential expression analysis revealed genes with treatment-associated expression changes. The candidate hub genes identified through the original integration of WGCNA and differential expression results were subsequently characterized using single-cell and spatial transcriptomic analyses. This multi-omics approach helps address the limitations of individual techniques. Bulk transcriptomics provides greater statistical power for identifying disease-associated genes but lacks cellular resolution. Single-cell RNA-seq reveals cellular heterogeneity but may miss rare cell populations due to limited sampling. Spatial transcriptomics preserves tissue context but generally has lower throughput. By integrating these approaches, we can identify candidate molecular signals supported across multiple levels of analysis.

The seven candidate hub genes identified in this study represent AD-associated molecular signals that warrant further investigation. CCR7 and MMP12, in particular, showed strong associations with immune- cell abundance scores and AD-related molecular features. Immunohistochemical assessment of CCR7 provided supportive evidence for the corresponding transcriptomic pattern; however, the present findings do not establish the biomarker performance or clinical utility of CCR7 or the other candidate genes.

Several limitations should be acknowledged. The study utilized publicly available datasets, which may have inherent biases related to sample collection, processing, and clinical characteristics. The largely cross-sectional nature of the study precludes firm conclusions about the causal relationship between gene expression and disease progression. The available sample size while analyze may not capture all sources of biological heterogeneity, particularly across different clinical and experimental settings. **Figure 7** presents four representative lesional sections; therefore, the illustrated spatial patterns are descriptive and should not be generalized to the complete dataset or interpreted as evidence of cellular co-localization, migration, or interaction. Although available analysis scripts and pySCENIC intermediate outputs are provided as **Supplementary Materials**, missing environment configurations and reference or intermediate files preclude full end-to-end reproducibility. Validation in independent cohorts with longitudinal clinical data would be valuable for determining the reproducibility and potential clinical utility of the identified candidate molecular signals. The treatment-associated analyses were limited by small groups, potential within-patient dependence from repeated sampling, and incomplete standardized patient-level response outcomes, precluding development or validation of a reliable treatment-response prediction model. Moreover, FDR-adjusted sensitivity analysis supported only MMP12 and RGS1 in the lesional-versus-non-lesional comparison, whereas none of the seven genes reached nominal or FDR-adjusted significance in the paired treatment analysis. The treatment-associated and complete seven-gene findings should therefore be interpreted as exploratory and require confirmation in independent datasets.

The single-cell and spatial transcriptomics data were obtained from patient cohorts different from those used for the bulk transcriptomic and microarray analyses, which may introduce batch effects and cohort-related heterogeneity. However, we applied consistent analytical principles and examined the concordance of our findings across multiple datasets to reduce the potential impact of these differences.

SCENIC inference is susceptible to false-positive regulons and does not demonstrate direct transcription-factor binding or causal regulation. The candidate regulons identified in this study require replication in independent datasets and validation using perturbation experiments and direct chromatin-based assays.

Immunohistochemical assessment was limited to one selected candidate hub gene, CCR7, and included only five independent biological samples per group because of antibody availability and limited experimental materials. In addition, the diffuse epidermal and stratum corneum immunoreactivity may include nonspecific background. Because co-localization with immune-cell markers was not performed, the precise cellular source and subcellular localization of CCR7 immunoreactivity could not be established. Complete original images from all IHC samples and the antibody dilution information were not available in the archived experimental records, which limits the reproducibility and independent evaluation of the immunohistochemical findings. Future studies should assess CCR7 and additional candidate hub genes, including GZMB, IL7R, and MMP12, using appropriate staining controls and cell-type-specific co-localization in larger, independent cohorts.

## 5 Conclusion

In conclusion, this exploratory multi-omics study identified seven exploratory candidate hub genes through nominal-*P*-value-based integration and characterized their associations with AD-related molecular features and immune-cell abundance patterns. FDR-adjusted sensitivity analysis supported MMP12 and RGS1 in the lesional-versus-non-lesional comparison but did not support the robustness of the complete seven-gene set or identify significant candidate-gene differences in the paired treatment analysis. Single-cell and spatial analyses further characterized their cell-type-associated expression patterns, computationally inferred regulon activity, and inferred spatial cell-population distributions. These findings provide a hypothesis-generating framework for understanding molecular heterogeneity in AD but do not establish diagnostic biomarkers, therapeutic targets, causal regulatory mechanisms, or predictors of treatment response. Future studies should prioritize independent cohort replication, functional validation, standardized spatial analyses, and evaluation using prespecified clinical outcomespractice.

## Data Availability

No new raw transcriptomic data were generated in this study. The public microarray, single-cell RNA-seq, and spatial transcriptomic datasets analyzed in this study are available in the Gene Expression Omnibus (GEO) under accession numbers GSE130588, GSE59294, GSE153760, GSE158432, and GSE197023. Individual relative CCR7 immunoreactivity measurements generated for the present study are provided in **Supplementary Table S2**. To facilitate reproducibility, the revised Methods provide the datasets, software and package versions, preprocessing procedures, quality-control criteria, statistical thresholds, and principal analytical steps used throughout the study. Available analysis scripts, including the code used for the paired treatment sensitivity analysis, are provided as **Supplementary Code S1**. Available pySCENIC intermediate outputs are provided as **Supplementary Data Sheet S1**. However, some original environment configurations, reference resources, and intermediate objects were unavailable; therefore, the deposited materials do not constitute a fully executable end-to-end workflow.
