# Supplementary material for: Integration of bulk and single-cell transcriptomics with spatial analysis identifies candidate hub genes and inferred regulatory programs in atopic dermatitis
Source: Front Immunol. 2026 Sep 30;17:1863607. doi: 10.3389/fimmu.2026.1863607 (PMC13661441; doi:10.3389/fimmu.2026.1863607)
Supplement: Supplementary file 1 [file SupplementaryFile1.docx]

Supplementary Table S1 summarizes the public transcriptomic datasets, sample composition, and analytical purposes. Supplementary Table S2 provides the individual relative CCR7 immunoreactivity measurements used in Figure 8B. Supplementary Table S3 summarizes the available sample-level metadata for the single-cell RNA-seq query cohort. In Supplementary Table S3, variables unavailable in the original source records are marked as ‘NA’ and were not imputed or inferred. Supplementary Table S4 provides nominal and FDR-adjusted statistical results for the seven exploratory candidate hub genes. Supplementary Table S5 lists the nine matched pre- and post-treatment sample pairs used in the paired sensitivity analysis. Supplementary Figure S1 shows the paired expression patterns of the seven candidate genes.

**Supplementary Table S1. Public transcriptomic datasets and sample information**

| **Dataset** | **Data type/**  **platform** | **Sample information** | **Analysis** |
| --- | --- | --- | --- |
| GSE130588 | Microarray;  Affymetrix Human Genome U133 Plus 2.0 Array (GPL570) | 124 lesional AD skin samples | WGCNA; clinical-trait correlation analysis; Gene Ontology enrichment analysis; immune-cell abundance estimation |
| GSE59294 | Microarray; Affymetrix Human Genome U133 Plus 2.0 Array (GPL570) | Lesional skin (LS, n = 16), non-lesional skin (NL, n = 7), and pre-/post-treatment samples | Differential expression analysis; immune-cell abundance estimation |
| GSE153760  And GSE158432 | Single-cell RNA sequencing | Combined query cohort comprising 66,846 cells from 25 samples: untreated AD (n = 9), dupilumab 16 weeks (n = 5), dupilumab 1 year (n = 4), and healthy controls (n = 7) | Reference mapping; cell-type annotation; cell-type-associated expression analysis; computational inference and comparison of regulon activity |
| GSE197023 | Spatial transcriptomics; 10x Genomics Visium | 20 skin samples: lesional AD (n = 7), non-lesional AD (n = 7), and healthy controls (n = 6) | Seurat processing; RCTD deconvolution; inference of spatial cell-population distributions |

AD, atopic dermatitis; LS, lesional skin; NL, non-lesional skin; WGCNA, weighted gene co-expression network analysis; RCTD, Robust Cell Type Decomposition. Sample counts refer to biological samples, whereas the reported cell count refers to individual single-cell transcriptomic profiles. Downstream single-cell analyses were restricted to blister-derived samples and subclusters containing more than 200 cells to reduce potential sampling-related bias and instability associated with very small subclusters. Sample-level demographic and clinical variables were reported only when available in the original public records.

**Supplementary Table S2. Individual quantitative measurements of CCR7 immunoreactivity**

| **Sample ID** | **Group** | **Relative CCR7 immunoreactivity** |
| --- | --- | --- |
| HC1 | Healthy control | 0.5681 |
| HC2 | Healthy control | 1.0654 |
| HC3 | Healthy control | 0.8297 |
| HC4 | Healthy control | 0.9917 |
| HC5 | Healthy control | 1.5451 |
| AD1 | Lesional AD | 3.5863 |
| AD2 | Lesional AD | 2.3732 |
| AD3 | Lesional AD | 1.9529 |
| AD4 | Lesional AD | 1.3760 |
| AD5 | Lesional AD | 1.2500 |

Relative CCR7 immunoreactivity was measured in five independent healthy-control skin samples and five independent lesional AD skin samples. Values are reported as provided in the available experimental data and correspond to the individual measurements used in Figure 8B. Group differences were assessed using the Mann–Whitney U test (*P* = 0.0317). The relative measurements do not represent an H-score or a count of positive cells per mm².

**Supplementary Table S3. Available sample-level metadata for the single-cell RNA-seq query cohort**

| **Dataset** | **GSM ID** | **Sample ID** | **Sample type** | **Age (years)** | **Sex** | **Race** | **Diagnosis** | **Group** | **EASI score** | **EASI reduction from baseline (%)** | **Total serum IgE (kU/L)** | **Blood eosinophils (%)** |
| --- | --- | --- | --- | --- | --- | --- | --- | --- | --- | --- | --- | --- |
| GSE153760 | GSM4653863 | HC1 | Blister | 42 | Female | Caucasian | Healthy control | Healthy control | NA | NA | NA | NA |
| GSE153760 | GSM4653864 | HC2 | Blister | 42 | Female | Caucasian | Healthy control | Healthy control | NA | NA | NA | NA |
| GSE153760 | GSM4653865 | HC3 | Blister | 47 | Female | Caucasian | Healthy control | Healthy control | NA | NA | NA | NA |
| GSE153760 | GSM4653866 | HC4 | Blister | 49 | Female | Caucasian | Healthy control | Healthy control | NA | NA | NA | NA |
| GSE153760 | GSM4653867 | HC5 | Blister | 39 | Male | Caucasian | Healthy control | Healthy control | NA | NA | NA | NA |
| GSE153760 | GSM4653868 | HC6 | Biopsy | 27 | Female | Caucasian | Healthy control | Healthy control | NA | NA | NA | NA |
| GSE153760 | GSM4653869 | HC7 | Biopsy | 42 | Female | Caucasian | Healthy control | Healthy control | NA | NA | NA | NA |
| GSE153760 | GSM4653855 | AD1 | Blister | 18 | Male | Caucasian | Atopic dermatitis | Untreated AD | 34.2 | 0 | >5000 | 14 |
| GSE153760 | GSM4653856 | AD2 | Blister | 19 | Female | Caucasian | Atopic dermatitis | Untreated AD | 44.6 | 0 | 8.7 | 5.8 |
| GSE153760 | GSM4653857 | AD3 | Blister | 34 | Male | Caucasian | Atopic dermatitis | Untreated AD | 44.7 | 0 | 1138 | 9.6 |
| GSE153760 | GSM4653858 | AD4 | Blister | 33 | Female | Caucasian | Atopic dermatitis | Untreated AD | 5.5 | 0 | 180 | 4.2 |
| GSE153760 | GSM4653859 | AD5 | Biopsy | 31 | Female | Caucasian | Atopic dermatitis | Untreated AD | 24.1 | NA | NA | NA |
| GSE153760 | GSM4653860 | AD6 | Biopsy | 22 | Male | Caucasian | Atopic dermatitis | Untreated AD | 28.1 | NA | NA | NA |
| GSE153760 | GSM4653861 | AD7 | Biopsy | 24 | Male | Caucasian | Atopic dermatitis | Untreated AD | 42.8 | NA | NA | NA |
| GSE153760 | GSM4653862 | AD8 | Biopsy | 42 | Male | Caucasian | Atopic dermatitis | Untreated AD | 46.5 | NA | NA | NA |
| GSE158432 | GSM4800173 | AD13 | Blister | 45 | Female | Caucasian | Atopic dermatitis | Untreated AD | 27.4 | 0 | NA | 9 |
| GSE158432 | GSM4800170 | AD10 | Blister | 35 | Male | Caucasian | Atopic dermatitis | Dupilumab 16 weeks | 15.9 | 64.4 | 169 | 5.7 |
| GSE158432 | GSM4800171 | AD11 | Blister | 19 | Male | Caucasian | Atopic dermatitis | Dupilumab 16 weeks | 10.1 | 70.5 | 1702 | 13 |
| GSE158432 | GSM4800172 | AD12 | Blister | 19 | Female | Caucasian | Atopic dermatitis | Dupilumab 16 weeks | 2.4 | 94.6 | NA | NA |
| GSE158432 | GSM4800177 | AD17 | Blister | 41 | Male | Caucasian | Atopic dermatitis | Dupilumab 16 weeks | 9.0 | 50.0 | 579 | 3.5 |
| GSE158432 | GSM4800178 | AD18 | Blister | 45 | Female | Caucasian | Atopic dermatitis | Dupilumab 16 weeks | 5.4 | 80.3 | 2.82 | 2.6 |
| GSE158432 | GSM4800174 | AD14 | Blister | 60 | Female | Caucasian | Atopic dermatitis | Dupilumab 1 year | 3.2 | 92.9 | 3729 | 2 |

The single-cell RNA-seq query cohort comprised 25 samples from GSE153760 and GSE158432: 7 healthy-control samples, 9 untreated AD samples, 5 samples collected after 16 weeks of dupilumab treatment, and 4 samples collected after 1 year of dupilumab treatment. Downstream cell-type analyses were restricted to 19 blister-derived samples to reduce potential sampling-related bias. The EASI score and percentage reduction from baseline are presented in separate columns. “NA” indicates that the corresponding information was not available in the source records and was not imputed or inferred. AD, atopic dermatitis; EASI, Eczema Area and Severity Index; HC, healthy control; IgE, immunoglobulin E.

**Supplementary Table S4. Sensitivity-analysis results for the seven exploratory candidate hub genes**

| **Gene** | **LS vs NL log2FC** | **LS vs NL nominal P** | **LS vs NL BH-adjusted P** | **LS vs NL nominal criterion** | **LS vs NL FDR criterion** | **Paired Pre − Post mean difference** | **Paired nominal P** | **Paired BH-adjusted P** | **Pre > Post** | **Paired FDR criterion** |
| --- | --- | --- | --- | --- | --- | --- | --- | --- | --- | --- |
| GZMB | 1.9815 | 0.003460 | 0.058329 | Yes | No | 0.9112 | 0.229749 | 0.661968 | Yes | No |
| CCR7 | 1.1979 | 0.010817 | 0.106899 | Yes | No | 0.1554 | 0.758946 | 0.920427 | Yes | No |
| GPR183 | 1.1567 | 0.014776 | 0.124113 | Yes | No | 0.7409 | 0.156414 | 0.608566 | Yes | No |
| MMP12 | 3.5090 | 0.000986 | 0.031983 | Yes | Yes | 1.6540 | 0.118790 | 0.584401 | Yes | No |
| IL7R | 1.1234 | 0.008198 | 0.092728 | Yes | No | 0.4797 | 0.292875 | 0.696688 | Yes | No |
| RGS1 | 1.8138 | 0.000384 | 0.019132 | Yes | Yes | 0.5816 | 0.243651 | 0.670167 | Yes | No |
| KLHDC7B | 1.1197 | 0.020450 | 0.146866 | Yes | No | 0.3467 | 0.361307 | 0.739121 | Yes | No |

Positive LS-versus-NL log2 fold-change values indicate higher expression in lesional skin. Positive paired pre-versus-post mean differences indicate higher expression before treatment. Benjamini–Hochberg-adjusted *P* values were calculated across all genes tested in each comparison. The original exploratory criterion was an absolute log2 fold change or mean paired expression difference greater than 1 with a nominal *P* value below 0.05. The FDR-adjusted sensitivity criterion used the corresponding effect-size threshold and an FDR below 0.05.

**Supplementary Table S5. Matched pre- and post-dupilumab lesional skin samples included in the paired treatment sensitivity analysis.**

| **Patient ID** | **Pre-treatment GSM** | **Post-treatment GSM** | **Treatment regimen** | **Tissue** |
| --- | --- | --- | --- | --- |
| 13C | GSM1432970 | GSM1432971 | C Dupilumab 300 mg | LS skin |
| 14C | GSM1432974 | GSM1432975 | C Dupilumab 300 mg | LS skin |
| 1B | GSM1432944 | GSM1432945 | B Dupilumab 150 mg | LS skin |
| 2B | GSM1432947 | GSM1432948 | B Dupilumab 150 mg | LS skin |
| 3B | GSM1432949 | GSM1432950 | B Dupilumab 150 mg | LS skin |
| 4B | GSM1432951 | GSM1432953 | B Dupilumab 150 mg | LS skin |
| 5B | GSM1432954 | GSM1432955 | B Dupilumab 150 mg | LS skin |
| 6B | GSM1432958 | GSM1432959 | B Dupilumab 150 mg | LS skin |
| 9C | GSM1432962 | GSM1432963 | C Dupilumab 300 mg | LS skin |

Nine patients with matched lesional skin samples collected before and after dupilumab treatment were included. Pre_GSM and Post_GSM indicate the GEO sample accession numbers for the corresponding pretreatment and post-treatment samples, respectively. Treatment doses are reported according to the available GSE59294 sample metadata.

**Supplementary Figure S1. Paired expression patterns of the seven exploratory candidate hub genes before and after dupilumab treatment.** Paired expression levels of GZMB, CCR7, GPR183, MMP12, IL7R, RGS1, and KLHDC7B are shown for nine patients with matched lesional skin samples collected before and after dupilumab treatment in GSE59294. Lines connect samples from the same patient. Nominal P values were calculated using two-sided paired t tests, and FDR values were obtained using Benjamini–Hochberg adjustment across all 21,344 tested genes.
